# Enhancing aphid resistance in horticultural crops: a breeding prospective

**DOI:** 10.1093/hr/uhae275

**Published:** 2024-09-28

**Authors:** Lili Zhang, Chaoyan Chen, Yao Li, Chunyu Suo, Wei Zhou, Xiaowei Liu, Yizhuo Deng, Hamza Sohail, Ziyi Li, Fang Liu, Xuehao Chen, Xiaodong Yang

**Affiliations:** College of Horticulture and Landscape Architecture, Yangzhou University, Yangzhou, Jiangsu 225009, China; College of Horticulture and Landscape Architecture, Yangzhou University, Yangzhou, Jiangsu 225009, China; College of Plant Protection, Yangzhou University, Yangzhou, Jiangsu 225009, China; College of Horticulture and Landscape Architecture, Yangzhou University, Yangzhou, Jiangsu 225009, China; College of Horticulture and Landscape Architecture, Yangzhou University, Yangzhou, Jiangsu 225009, China; College of Horticulture and Landscape Architecture, Yangzhou University, Yangzhou, Jiangsu 225009, China; College of Horticulture and Landscape Architecture, Yangzhou University, Yangzhou, Jiangsu 225009, China; College of Horticulture and Landscape Architecture, Yangzhou University, Yangzhou, Jiangsu 225009, China; College of Horticulture and Landscape Architecture, Yangzhou University, Yangzhou, Jiangsu 225009, China; College of Plant Protection, Yangzhou University, Yangzhou, Jiangsu 225009, China; College of Horticulture and Landscape Architecture, Yangzhou University, Yangzhou, Jiangsu 225009, China; Joint International Research Laboratory of Agriculture and Agri-Product Safety, Ministry of Education of China, Yangzhou University, Yangzhou, Jiangsu 225009, China; College of Horticulture and Landscape Architecture, Yangzhou University, Yangzhou, Jiangsu 225009, China

## Abstract

Increasing agricultural losses caused by insect infestations are a significant problem, so it is important to generate pest-resistant crop varieties to address this issue. Several reviews have examined aphid–plant interactions from an entomological perspective. However, few have specifically focused on plant resistance mechanisms to aphids and their applications in breeding for aphid resistance. In this review, we first outline the types of resistance to aphids in plants, namely antixenosis, tolerance (cell wall lignification, resistance proteins), and antibiosis, and we discuss strategies based on each of these resistance mechanisms to generate plant varieties with improved resistance. We then outline research on the complex interactions amongst plants, viruses, and aphids, and discuss how aspects of these interactions can be exploited to improve aphid resistance. A deeper understanding of the epigenetic mechanisms related to induced resistance, i.e. the phenomenon where plants become more resistant to a stress they have encountered previously, may allow for its exploitation in breeding for aphid resistance. Wild relatives of crop plants serve as important sources of resistance traits. Genes related to these traits can be introduced into cultivated crop varieties by breeding or genetic modification, and *de novo* domestication of wild varieties can be used to exploit multiple excellent characteristics, including aphid resistance. Finally, we discuss the use of molecular design breeding, genomic data, and gene editing to generate new aphid-resistant, high-quality crop varieties.

## Introduction

Sustainable crop production is necessary for global food security under uncertain environmental conditions. With global climate change, many pests, including aphids, are becoming a serious problem, leading to significant crop losses. Global yields of key cereal crops are reduced by 5% to 20% as a result of pest damage at present, and this percentage is expected to increase by 10% to 25% for each degree Celsius rise in global temperature attributed to global warming [1].

During evolution, plants have evolved various strategies to resist or tolerate aphids. Waxes, trichomes, and the cuticle provide physical barriers, and the emission of volatile compounds can deter aphids [2, 3]. Upon feeding by aphids, plants mount various defence strategies; some plants are able to produce compounds that poison attacking insects, e.g. betulin is produced by peach (*Prunus persica*) in response to aphid attack [4]. Recent research indicates that induced resistance, i.e. the phenomenon where plants are better able to tolerate a stress they have encountered before, is under epigenetic control, so there is potential to generate resistant populations that have inherited this pattern of genetic control, and are therefore more stress resistant [5].

There is considerable potential to exploit these natural strategies for pest resistance to produce aphid-resistant crop varieties [6]. Conventional breeding techniques have played an important role in increasing crop resistance to various pests, but the continuing evolution of aphid populations and their capacity to adapt to existing plant defences necessitates ongoing research and development. In this review, we discuss progress in research on the mechanisms of aphid resistance in plants, and how these mechanisms can be exploited to produce resistant crop varieties using traditional breeding or new molecular methods.

Recent progress in molecular biology, genomics, and biotechnology has provided new tools for combating aphid infestations [7]. For example, quantitative trait loci (QTLs) mapping has contributed to the breeding of aphid-resistant crops. There have been major advances in breeding for aphid resistance with the identification of numerous QTLs and genes related to aphid resistance. QTL mapping and transcriptomic analyses have identified genetic regions and candidate genes related to aphid resistance in maize (*Zea mays*), cotton (*Gossypium hirsutum*), soybean (*Glycine max*), and cucumber (*Cucumis sativus*), as discussed later in this review [8, 9]. The candidate genes encode products with a range of functions, such as lignin synthesis and pathogen recognition [10]. Another example is the use of a multi-target RNAi strategy based on sugarcane yellow leaf virus to improve aphid resistance in sugarcane [11]. Transgenic plants expressing genes related to resistance have been produced, and they show increased repellency to aphids in choice tests. These examples highlight the use of transgenic technology to breed aphid-resistant crop varieties.

In this review, we outline recent advances in breeding for aphid resistance in crop plants, highlighting new prospects and potential breakthroughs in the field. The aim of current research is to design crops with robust aphid resistance. This is underpinned by studies on the molecular mechanisms of plant–aphid interactions, so that these interactions can be exploited to improve resistance. Breeding aphid-resistant crops will reduce the need for chemical pesticides and promote sustainable agriculture practises. In the following pages, we provide an overview of the progress and future perspectives for breeding aphid-resistant plants as a step towards sustainable crop production.

## Natural mechanisms of aphid resistance: antixenosis, tolerance, and antibiosis

**Figure 1:**
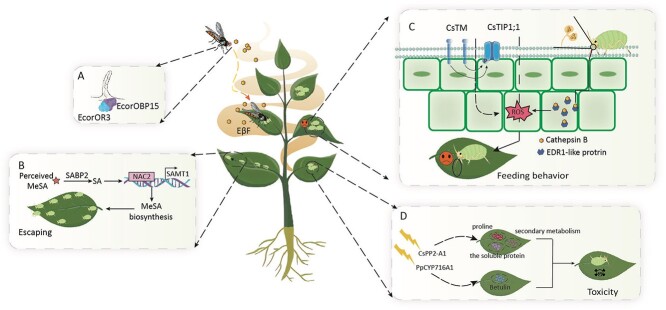
Three defence mechanisms of plants against aphids: antixenosis, tolerance, and antibiosis. Fig. A and B tells the aphid’s antixenosis mechanism, Fig. B tells the aphid’s tolerance mechanism, and Fig. C tells the aphid’s antibiotic mechanism. (A–B) Plants attacks by aphids emit (E)-*β*-farnesene (E*β*F), which can be sensed by neurons of adults of aphid predator hoverfly *E. corollae*. The specific sensing mechanism is that the odourant receptor EcorOR3 in adult worms binds to the odourant-binding protein EcorOBP15 and accurately identifies aphid-infested plants by sensing the odour of E*β*F. Adults of aphid predator hoverfly *E. corollae* will fly to the surface of an aphid-infested plant to eat aphids and improve the plant’s ability to avoid aphids [17]. MeSA releases by plants can be sensed by the *SABP2* receptor in plants and converts to SA, which triggers the NAC2-SAMT1 mechanism to synthesize more MeSA. When aphids sense MeSA, they will escape the plant [16]. (C) CsTM interacts with the water channel protein CsTIP1;1 to regulate trichome morphology and reduce aphid attractiveness and fitness by modulating hydrogen peroxide. CathB3, an inducible protein in the saliva of peach aphid, is secreted into tobacco and interacts with plant EDR1-like proteins, triggering a burst of ROS in the phloem and inhibiting phloem feeding and colonization by aphid [23]. (D) Overexpression of *CsPP2-A1* in cucumber increased the levels of secondary metabolites (PAL, PPO, the phenols, and flavonoids), proline, and the soluble proteins, leading to aphid death. The *PpCYP716A1* gene in peach regulates the synthesis of the metabolite betulin, which is directly toxic to green peach aphids [33].

During evolution, plants have evolved a multitude of intricate defence mechanisms to fend off aphids. Plant defence mechanisms against aphids include antixenosis, tolerance, and antibiosis [12] (Fig. 1).

### Antixenosis mechanisms involved in defence against aphids

Plants have developed various antixenosis mechanisms to protect themselves from aphids. These include physical structures like trichomes, waxes, and the cuticle on the leaf surface, and volatile substances such as ethylene (ET), phenols, sesquiterpenes, and methyl salicylate (MeSA) that are released from plant tissues [2, 3]. In *Physcomitrium patens*, the AP2/ERF transcriptional activator encoded by *PpWIN1* promotes the biosynthesis of cuticle and cuticular waxes, and it had the same function when expressed in *Arabidopsis* [13]. Some plants produce volatile organic compounds (VOCs) such as sesquiterpenes, MeSA, and (E)-*β*-farnesene (E*β*F), as a defence against insect attack [14]. In tomato, the mixture of monoterpenes and a small amount of sesquiterpenes produced by the glandular trichomes was shown to affect the lifespan and behavioural selection of potato aphid (*Macrosiphum euphorbiae*). The sesquiterpenes produced by the monoterpene synthase TPS12 were shown to contribute to resistance to *M. euphorbiae* [15]. Other plants release MeSA as a response to insect attack. For example, when aphids feed on tobacco (*Nicotiana tabacum*), the plants release MeSA, which is subsequently recognized by the receptor protein SABP2 in neighbouring tobacco plants and transformed into salicylic acid (SA). In turn, SA activates NAC2 and SAMT1 to enhance MeSA synthesis, resulting in a considerable increase in the aphid repellency of tobacco plants that have not yet been attacked [16] (Fig. 1B). An example of the exploitation of this mechanism to improve aphid resistance is the introduction of the *sesquiterpene synthase 2* (*SsT2*) gene from *Solanum habrochaites* into cultivated tomato. This resulted in increased sesquiterpene content and enhanced repellency to aphids in the transgenic lines [2].

The release of odourant molecules to attract aphid predators is another effective antixenosis mechanism. An example of this is the aphid predator *Eupeodes corollae*, which has an effective E*β*F-mediated localization mechanism in its antennae. The olfactory receptor protein EcorOR3 binds to the olfactory-binding protein EcorOBP15, and this allows it to locate its prey on plants emitting E*β*F. This interaction has been exploited to produce plants with increased repellency to aphid pests [17] (Fig. 1A). Transgenic *Medicago sativa* plants expressing an E*β*F synthase gene showed increased E*β*F production, and exhibited considerably higher repellency against pea aphid (*Acyrthosiphon pisum* Harris) [18].

All of the antixenosis mechanisms mentioned above significantly reduce aphid invasion and damage [18]. Therefore, they have considerable potential for use in breeding programmes to generate new aphid-resistant crop varieties.

### Plant tolerance mechanisms

Plants have evolved several mechanisms of tolerance, including increased contents of substances related to resistance, and activation of the immune system to resist repeated pes aphid attacks [19]. For example, in chrysanthemum attacked by aphids, many MYB transcription factors enhance lignin production, resulting in thickened cell walls that create a physical and chemical barrier against aphid invasion [20]. In cucumber, a melatonin treatment triggered an anti-aphid signalling system, leading to a rise in jasmonic acid (JA) and flavonoid contents. This attenuated aphid reproduction and improved the aphid resistance of cucumber plants [21]. Treatment of *Arabidopsis* with oligogalacturonides resulted in callose deposition, the accumulation of reactive oxygen species (ROS), and cell wall thickening. These changes diminished the intensity of aphid feeding [22]. In cucumber (*C. sativus* L.), *CsTM* was identified as a gene affecting trichome morphology, with the knockout mutant of *CsTM* showing impaired epidermal trichome development and being almost hairless. Further research indicated that CsTM is a C-lectin receptor-like kinase that interacts with the water channel protein CsTIP1;1 to regulate trichome morphology via a mechanism involving H_2_O_2_, Ca^2+^, and cellulose synthase. By controlling intracellular signalling and the phenotype of trichomes, *CsTM* increases the resistance of cucumber plants to cotton aphid (*Aphis gossypii* Glover) [23] (Fig. 1C).

During feeding, aphids release salivary proteins into the plant. This signal activates pattern-induced immune responses and effector-triggered immunity in the attacked plants. These immune responses activate ROS and Ca^2+^ signalling pathways, which in turn enhance immune defences, including the kinase cascade response [24]. The elicitor protein CathB3, which is secreted by green peach aphid (*Myzus persicae*), interacts with the EDR1-like protein in tobacco. This triggers a burst of ROS in the phloem, leading to inhibition of aphid feeding and restricted colonization of the phloem [25] (Fig. 1C). The *Arabidopsis* R gene *SLI1* encodes a small heat-shock protein SLI1, which is involved in resistance against sucking insects. Analyses of *Arabidopsis sli1* mutants confirmed that this protein confers broad-spectrum resistance to phloem-feeding insects, including peach aphid [26]. Aphid effector proteins cause NLR proteins in the host to elicit an immunological response, resulting in the disruption of aphid nutrition, lower aphid lifespan, and reduced aphid fecundity [27]. In tomato, the transcription factor *SlMYB75* is related to increased trichome coverage and trichome hardness, both of which are characters that may slow the rate of aphid infestation and reduce aphid adaptation [28]. In addition, overexpression of *SlSERK1*, which encodes a receptor in the plant cell membrane involved in the immune response, led to a significant decrease in the lifespan and reproductive capacity of aphids. *SlSERK1* overexpression in tomato also increased the effectiveness of Mi-1.2-mediated immune responses and stimulated the expression of *SlWRKY72b*, which encodes a transcription factor related to resistance [29].

The results of all of these studies indicate that plants can strengthen their resistance to aphids by increasing the amounts of certain substances and/or proteins, and that overexpression of genes encoding crucial immunomodulatory factors can increase aphid resistance.

### Antibiosis mechanisms

Antibiosis is when plants produce poisonous secondary metabolites or lectins that directly interfere with the normal physiological processes of aphids, or cause insect death [30]. An example of antibiosis is the synthesis of betulin, catalysed by *PpCYP716A1* gene, in peach as a response to green peach aphid attack. Betulin has strong insecticidal activity and exhibits direct toxic effects on green peach aphid [4] (Fig. 1D). When *Arabidopsis* plants are attacked by the aphid *Pieris rapae* or mechanically damaged, thioredoxins are degraded into isothiocyanates that are toxic to herbivores [31]. Pepper (*Capsicum annuum* L.) plants attacked by peach aphid produce metabolites with toxic and repellent properties. These metabolites include thioglucosides, alkaloids, terpenoids, phenolics, and protease inhibitors [32].

Antibiosis mechanisms have been exploited to produce aphid-resistant transgenic plants. For example, the upregulation of the lectin-encoding gene *CsPP2-A1* in cucumber resulted in the stabilization of ROS levels in the plant, and the accumulation of secondary metabolites via increased activities of key enzymes involved in phenol and flavonoid synthesis (phenylalanine ammonia lyase and polyphenol oxidase) [33] (Fig. 1D). Ultimately, these changes resulted in a substantial decline in aphid reproduction and a substantial increase in aphid mortality. The introduction of the snowdrop lectin protein ASGNA into *Arabidopsis* and cotton plants resulted in the inhibition of aphid growth and reproduction, leading to a considerable decrease in the aphid survival rate. The presence of snowdrop lectin protein greatly enhanced the resistance of *Arabidopsis* and cotton plants to aphids [34]. The results of these studies demonstrate that antibiosis mechanisms can have substantial inhibitory or even deadly or effects on aphids.

To summarize, plant defences against aphids are intricate systems representing a variety of strategies with multiple levels and participants. These encompass antixenosis mechanisms, which deter aphids; and tolerance mechanisms, which enhance resistance after aphid attack. These systems work together to prevent harm to the plant. Antibiosis is a very efficient defence system because it kills the attacking insects. This mechanism has great potential for crop protection in an environmentally friendly manner. The production of plants that deter insects, are resistant insect attack, and/or are toxic to insects will result in reduced use of chemical pesticides, which will not only decrease the environmental impact of agricultural production, but also enhance the quality and quantity of crops produced. Therefore, all of these strategies can be exploited for sustainable growth in agriculture.

## Interactions amongst plants, aphids, and viruses

**Figure 2:**
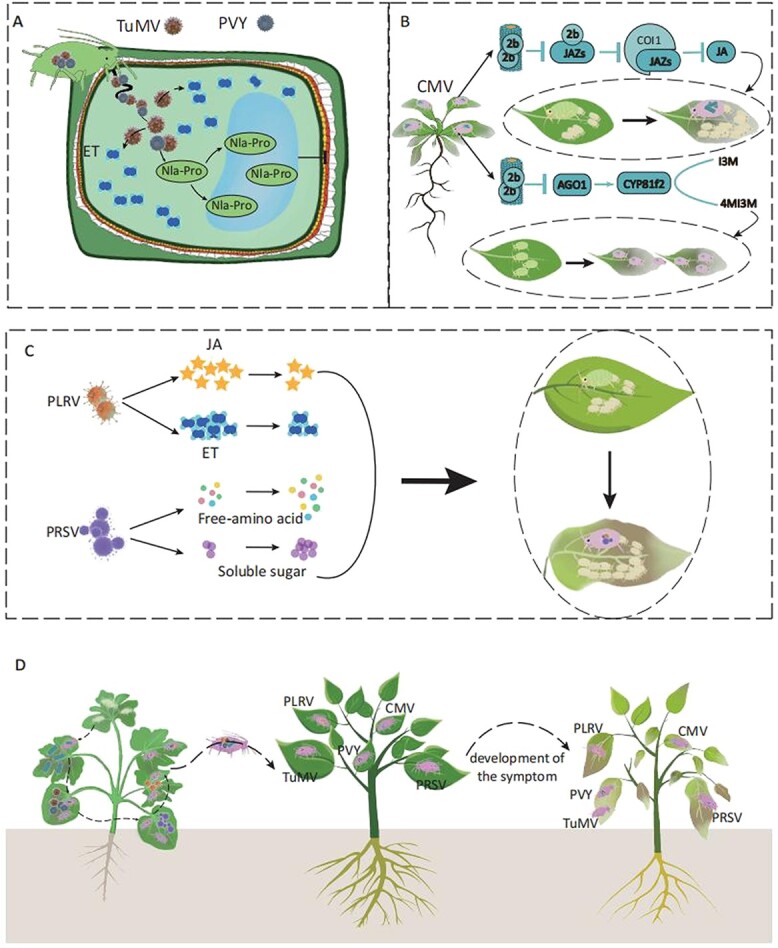
Interaction mechanism between viruses, aphids, and plants. (A) Aphids carrying a single TuMV protein, nuclear inclusion a-protease domain (Nla-Pro) transmit the virus to the plant via stylet when they ingest *Arabidopsis* or tobacco. Moreover, the TuMV protein Nla-Pro induces the production of ET, which is necessary for TuMV to inhibit the resistance of *Arabidopsis* to green peach aphids by suppressing callose deposition. When aphids act as vectors for TuMV and PVY transmission and transmit the virus to the host plant, changes in protein localization occur, i.e. when the TuMV protein Nla-Pro locates in the vacuole, it increases the proliferation of the vector aphids on the plant and suppresses callose deposition [49]. (B) When aphids carrying CMV virus feed on plants, the 2b protein of the virus enters the plant, binds to JA inhibitory protein JAZs, inhibits the binding of JAZs to JA signal receptor COI1, inhibits the ubiquitination degradation of JAZs, and thus inhibits JA signalling pathway, making the plant more attractive to aphids and conducive to aphid reproduction compared to aphids settling on plants that are not infected with the virus (dotted line of the change in aphid behaviour). The virus 2b protein can also directly bind to and inhibit *AGO1*, which positively regulates *CYP81f2* expression. *CYP81f2* catalyses the synthesis of 4MI3M from the aphid deterrent I3M, which promotes aphid dispersal and facilitates virus transmission compared to aphids settling on plants that are not infected with the virus (dotted line of the change in aphid behaviour) [52, 53]. (C) PLRV-carrying aphids infected plants, weakened the plant’s defence response of JA and ET, and increased the aphids’ ability to reproduce. When aphids carrying PRSV virus infect plants, it increases the accumulation of free amino acids and soluble sugars in the plant, which provide nutrients for the growth and reproduction of aphids; thus it prolongs the feeding time of aphids and enhances the adaptability of aphids to plants. The behaviour of aphids on plants infected with PLRV and PRSV changed as shown in dotted lines, compared to aphids settling on plants that were not infected with the virus [54, 55]. (D) The plant is infected with the above five viruses, including PVY, TuMV, CMV, PLRV, and PRSV. After the aphid sucks the plant, it will move the virus to other plants, which will become infected with these five viruses and develop the symptom of yellow and wilt [36–38]. TuMV: turnip mosaic virus; PVY: potato virus Y; CMV: cucumber mosaic virus; PLRV: potato leafroll virus, PRSV: papaya ringspot virus.

Aphids can transmit plant viruses to their hosts. Viruses negatively affect their host plants, causing yellowing and other symptoms. These symptoms can affect aphids’ selection behaviour for their host plants [35] (Fig. 2). For example, virus-derived small interfering RNAs from viruses like cucumber mosaic virus (CMV) and barley yellow dwarf virus target genes involved in chlorophyll synthesis in their hosts, leading to yellowing symptoms [36, 37]. Leaf yellowing is one strategy by which plant viruses attract aphids to ensure their transmission. Yellowed leaves do not produce insect-deterring odour compounds, so insects preferentially choose yellow plant tissues [38] (Fig. 2D).

### Aphid-transmitted plant viruses alter the emission of VOCs

The emission of VOCs from host plants can be qualitatively or quantitatively altered by plant viruses that are spread by aphids [39], and this can influence aphids’ selection behaviour towards the host plant. In general, plants infected by viruses are more heavily preyed upon by aphids [40]. Moreover, aphids’ preference for feeding on healthy or infected plants is affected by the infection status of the host [41]. That is, non-viruliferous aphids prefer to feed on infected plants, whereas viruliferous aphids typically feed on healthy, non-infected plants [42].

In tobacco plants infected with tomato yellow leaf curl virus, the synthesis of terpenoid compounds is inhibited, leading to a reduction in resistance to aphids [43]. Both black raspberry necrosis virus and raspberry leaf mottle virus cause infected raspberry plants to release VOCs that attract the aphid *Amphorophora idaei* Borne [44]. Similarly, zucchini (*Cucurbita pepo*) plants infected with CMV show increased synthesis of VOCs, making the plants more attractive to aphids. The VOCs attract aphids to the infected plants, where they acquire the virus whilst feeding. Then, the aphids that have fed on CMV-infected plants find other hosts and transmit the virus to healthy plants [45].

The VOCs produced by virus-infected host plants not only attract aphids, but also tend to attract pollinating or predatory insects. For instance, tomato plants infected with CMV become more attractive to bumblebees for pollination [46], and pepper plants infected with CMV or potato virus Y become more attractive to aphid parasitoids [47]. This creates a new ecological balance by acting as a compensatory mechanism for plants that have recovered from viral infection and have decreased defences.

### Aphid-transmitted viruses induce changes in plant signalling pathways

The infection of host plants by aphid-transmitted plant viruses can affect plant signalling pathways, resulting in increased aphid fecundity and survival to facilitate virus spread.

The turnip mosaic virus protein Nla-Pro inhibits aphid-induced callose formation in an ET-dependent manner, thereby reducing the plant defence response [48]. Nla-Pro is transmitted to the host plant by aphids. The reproductive ability of aphids in the host plant is affected by the localization of Nla-Pro, i.e. aphids are able to grow and reproduce only after Nla-Pro is relocated to the vacuole. A similar mechanism has been detected in the potato (*Solanum tuberosum*)–*M. persicae* (green peach aphid)–potato virus Y interaction [49] (Fig. 2A).

After infection by CMV in *Arabidopsis*, the virus-encoded 2b protein directly interacts with JA repressor proteins, JASMONATE ZIM-domains (JAZs). This association prevents JAZs from being degraded by ubiquitination by weakening the binding between JAZs and the JA signalling receptor COI1 (Fig. 2B). Consequently, CMV infection suppresses the JA signalling pathway, increasing host plant attractiveness to aphids to aid the spread of the virus [50, 51]. Furthermore, the CMV-encoded 2b protein inhibits AGO1, a transcription factor that promotes the expression of *CYP81f2*. This gene encodes an enzyme that synthesizes the aphid-repelling compound 4-methoxy-indol3yl-methylglucosinolate. These mechanisms attract aphids to the infected plants, and facilitate their migration to other plants, thereby spreading the CMV virus [52, 53] (Fig. 2 B).

The increased attractiveness and reproductive capacity of aphids on potato plants infected with potato leafroll virus are associated with a decrease in JA and ET signalling [54] (Fig. 2C). Papaya ring spot virus infection in pumpkin (*C. pepo*) makes the plants more attractive to cotton aphids by increasing the contents of nutrients including free amino acids and soluble carbohydrates [55] (Fig. 2C).

In summary, the interaction between viruses and vectors reduces plant defence responses and/or increases the attractiveness of the plant to the insect vector to favour survival of the virus. In general, plant viruses are transferred by aphids, proliferate within host cells, and alter plant immune responses, and insects exploit the virus to reduce plant defence responses. The relationships amongst plants, viruses, and insects are complicated, and require further research.

## Introducing excellent traits from wild resources through hybridization or *de novo* domestication

Compared with cultivated crops, their wild and semi-wild relatives typically exhibit broader genetic diversity, so they often harbour resistance traits distinct from those in cultivated crops [56].

### Introducing excellent traits of wild resources through hybridization

Excellent traits from wild resources can be introduced into cultivated varieties by hybridization. An example of this is the trigenomic hybridization of chrysanthemum, which involved the crossing of two intergeneric F_1_ hybrids (*Chrysanthemum grandiflorum* × *Artemisia vulgaris* and *Chrysanthemum crassum* × *Crossostephium chinense*). This trigenomic hybridization combined multiple resistance traits from wild relatives, and the hybrid offspring showed increased resistance to aphids and salt tolerance. The hybrid offspring inherited beneficial genes from different parents, including genes encoding bioactive components from *A. vulgaris*; and genes involved in sodium (Na^+^) and potassium (K^+^) ion partitioning from *C. crassum* and *C. chinense*. The products of these genes improved selective transport amongst different organs, thereby preventing harmful effects and protecting photosynthetic organs [57]. The results of that study demonstrate the effectiveness of introducing beneficial traits from wild resources into cultivated lines to dramatically improve their stress resistance.

Another example is the introduction of the ability to produce VOCs to chrysanthemum. *Chrysanthemum aromaticum*, an important wild chrysanthemum resource, exhibits strong aphid resistance. This is because it produces a series of VOCs, including cis-4-thujanol, which are emitted from the leaves and have a repellent effect on aphids. Upon hybridizing this wild chrysanthemum with a common cultivated chrysanthemum, *Chrysanthemum nankingense*, resistance traits against aphids were transmitted to some of the offspring. Some of the progeny of the *C. aromaticum* × cultivated chrysanthemum cross were able to produce these VOCs, and therefore, they showed increased resistance to chrysanthemum aphid (*Macrosiphoniella sanborni*), one of the most damaging aphids of cultivated chrysanthemum [58].

Crossing wild ancestors with domesticated crops can potentially enhance their resistance against insects. Compared with cultivated potato, wild potato has superior resistance and produces more VOCs, which play important roles in plant defence such as repelling aphids, attracting natural enemies, and activating plant defence mechanisms. The amount of VOCs released by wild potatoes is significantly higher than those released by cultivated potatoes. Accordingly, the survival and reproduction of green peach aphid were found to be markedly reduced in the wild potato varieties (CGN18333, CGN22718, CGN23072) compared with those of the cultivated variety Desiree. Olfactometer bioassays gave comparable results, with wild varieties repelling adult green peach aphids. Conversely, the volatile compounds emitted by the wild potato line CGN18333 attracted the parasitic wasp *Diaeretiella rapae* (M’Intosh), resulting in decreased survival and reproduction of green peach aphid. The VOCs emitted by wild potato include *β*-caryophyllene, E*β*F, trans-*α*-bergamotene, _D_-limonene, (*E*,*E*)-4,8,12-trimethyl-1,3,7,11-tridecatetraene, and *p*-methylbenzyl alcohol, all of which influence the behaviour of green peach aphid and parasitic wasps [59].

Although hybridization is one of the most useful strategies to introduce traits from wild resources into cultivated resources, incompatibility is an issue for many plant species, and this limits the feasibility of this strategy [60]. Research on the mechanisms of self-incompatibility is essential to broaden the application of hybridization in generating aphid-resistant crop varieties.

Studies have shown that self-incompatibility in the Brassicaceae can be eliminated by decreasing the ROS content in the stigma [61]. Further research revealed the significant role of ROS in interspecific isolation between *Brassica rapa* and *Barbarea vulgaris* [62]. The breaking of interspecific isolation was made possible by decreasing the ROS content in the stigma or inhibiting the expression of genes related to ROS in the stigma. Breaking of self-incompatibility may allow for the transfer of genes related to pest resistance from *B. vulgaris* to *B. rapa* [63].

The degree of self-incompatibility between wild predecessors and cultivated varieties has grown during evolutionary history, and it restricts the integration of genes related to aphid resistance from semi-wild or wild resources into cultivated plant varieties. Breaking self-incompatibility is a critical technique to incorporate aphid-resistant genes from wild or semi-wild resources into cultivated cultivars to improve aphid resistance, reduce crop damage, and decrease reliance on pesticides. Some wild and semi-wild resources have beneficial traits distinct from those of cultivated resources, such as resistance to pests, diseases, drought, or salinity [64]. Through genetic improvement and selection, attempts can be made to improve unfavourable traits of wild resources to make them more suitable for agriculture and food production.

### Using *de novo* domestication to introduce aphid resistance characters from wild resources

**Figure 3:**
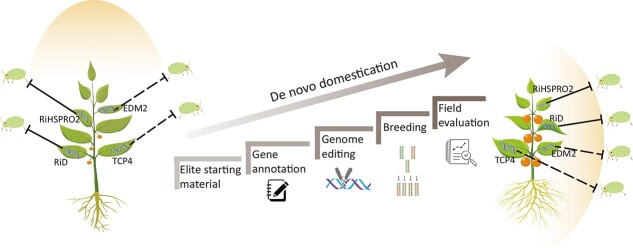
Process of *de novo* domestication of horticultural crops. Wild resources contain resistance genes to aphids. The figure shows the process of domesticating wild resources into conventional cultivars. (1) Select appropriate wild resource materials. (2) Gene annotation. Because most wild resource materials are very different from conventional cultivated species, their genomic information is unknown. Therefore, it is necessary to re-annotate the genomes of wild resources before domestication. (3) Editing of genes related to domestication. An effective editing system for wild resources was established to edit the genes of unfavourable traits in some wild resources. (4) Integrate favourable traits of the successfully edited lines and eventually use them in field breeding. (5) Assessment of plants planted in the field, including growth potential, aphid resistance, and yield-related traits. The solid line in the figure represents a protein-coding gene identified from the wild crucifer *R. indica*, which has been confirmed to have anti-aphid effects when introduced into *B. juncea*. In contrast, the dotted line indicates candidate genes with potential anti-aphid properties identified from a *de novo* transcriptome analysis. These candidate genes include *RiD* (*R. indica defensin*)[71], *RiHSPRO2* (*R. indica glutathione S-transferase*) [70], *glutaredoxin* [73], *EDM2* (*Enhanced Downy Mildew 2*) [73], and *TCP4* (*TCP family transcription factor 4*) [73].

*De novo* domestication is a strategy to improve wild resources. The aim of *de novo* domestication is to utilize the beneficial traits of wild species and gradually improve their unfavourable traits so that they are suitable for cultivation and meet the needs of consumers [65, 66] (Fig. 3).

*De novo* domestication can address issues that may arise when using conventional hybridization and self-breeding methods to improve crops, including reduced adaptability and the loss of genetic diversity. As a significant horticultural crop, the tomato’s genetic background and physiological characteristics make it an ideal model for studying crop ideotypes and the *de novo* domestication of wild relatives. By leveraging genome editing technology, the genetic makeup of crops can be precisely modified to enhance traits such as yield, quality, or disease resistance. This enables the cultivation of crop varieties with superior agronomic characteristics, tailored to meet specific agricultural needs [67]. For example, multiple CRISPR-Cas9 editing techniques were used to edit four stress-tolerant wild tomato varieties, introducing beneficial traits related to plant morphology, flower and fruit yield, and ascorbic acid synthesis. The offspring of the edited plants no longer exhibited wild tomato characteristics, but displayed domesticated features whilst retaining the superior stress resistance traits of the parent plant [68]. Another example is the use of gene-editing technology to edit six crucial sites in modern tomato varieties related to yield and productivity, successfully achieving re-domestication of the wild tomato variety *Solanum pimpinellifolium*. Compared with the unedited wild variety, the edited plants showed a 3-fold increase in fruit size and a tenfold increase in quantity. Notably, the accumulation of fruit lycopene was increased by 500% compared with that of widely cultivated *Solanum lycopersicum* varieties [69].

Research on *de novo* domestication is relatively limited, primarily because the genomes of wild resources differ significantly from those of cultivated varieties. A prerequisite for studying *de novo* domestication of wild resources is to assemble the wild species’ genomes and identify insect resistance genes. Transgenic *Brassica juncea* plant lines overexpressing selected aphid-induced genes from *Rorippa indica* (L.) Hiern [a plant defensin (RiD) and a nematode resistance protein (RiHSPRO2)] showed enhanced aphid tolerance [70, 71]. Previous studies have shown that cultivated Brassica, a member of the crucifer family, lacks resistance traits. However, through *de novo* transcriptome assembly and analysis, it has been discovered that *R. indica* (L.) Hiern possesses candidate resistance genes against *Lipaphis erysimi* (L.) Kaltenbach. Including *TCP4* (*AT3G15030*) and *EDM2* (*AT5G55390*), which are related to adverse stress response and SA-dependent reactive oxygen. These genes have the potential to be introduced into cultivated crops using genetic engineering techniques, thereby accelerating the process of *de novo* domestication and fostering the development of new varieties with enhanced insect resistance. This not only helps elucidate the resistance mechanisms of wild mustard against mustard aphids but also provides essential genetic resources and scientific evidence for *de novo* domestication. Ultimately, this approach holds significant potential for reducing pesticide use, protecting the environment, and increasing crop yields [72, 73].

Rice is one of the main crop plants used in *de novo* domestication research. To meet the demands of consumers and address issues with modern agriculture and food production, wild rice resources can be improved and new rice varieties developed by gene editing and genetic engineering technologies. Compared with traditional diploid cultivated rice, tetraploid rice exhibits advantages in terms of grain shape, weight, leaf area, and panicle length. Polyploid rice has useful characters such as wide genetic buffering, vigorous growth, and environmental adaptability. However, finding a feasible way to achieve polyploidisation has been challenging. Researchers identified *Oryza alta* as a polyploid genotype (CCDD) from screening a tetraploid wild rice library. This genotype, which was named ‘Polyploid rice 1 (PPR1)’, shows strong resistance to pests stresses and good biomass characteristics. Two critical resources have been established for this genotype: an efficient tissue culture, transformation, and genome-editing system; and a high-quality genome assembly divided into two subgenomes. Utilizing these resources, six important agronomic traits of *O. alta* were successfully improved, offering hope for polyploid rice to become a new staple food globally [74].

These discoveries provide new avenues for utilizing the genetic diversity present in wild plants in molecular breeding programmes. Through gene editing, researchers can create new crop varieties with increased resistance and productivity, incorporating beneficial traits from wild species whilst retaining agricultural advantages. This holds potential importance for future agriculture and food production. Research on *de novo* domestication of wild resources for aphid resistance is scarce, but this process is critical in breeding for aphid resistance. It provides breeders with abundant genetic resources, allowing them to develop new varieties with greater resistance and environmental adaptability, ultimately increasing crop outputs and quality whilst minimizing insect damage.

## Role of plant-induced resistance in breeding for aphid resistance

Plant-induced resistance is a significant example of phenotypic plasticity, where plants actively enhance their resistance to external stressors by building upon their own immunological responses [5]. This phenomenon is characterized by an increased ability to adapt and recover when exposed to the same stressor again, after the original contact and recovery. Induced resistance in plant-biotic stress interactions is driven by molecular regulatory mechanisms and genetic patterns. Numerous studies have detected strong links between plant-induced resistance and epigenetic variables, such as DNA methylation.

Induced resistance extends to a variety of pathogens and pests, including aphids. Early observations noted a 30% reduction in aphid population in cotton plants previously infested with aphids [75] and a 50% decrease in the reproductive capacity of bird cherry-oat aphid (*Rhopalosiphum padi* L.) on wheat seedlings previously infested with the same aphid [76]. Studies on the mechanisms of induced resistance in peach trees revealed that certain genotypic variations influenced resistance to peach aphid [77]. The mechanisms underlying induced resistance to aphids in plants are complex and involve interactions amongst plant secondary metabolites, hormonal signalling, and nutritional factors [75].

Plants respond to environmental stress by triggering resistance mechanisms, which not only improve the adaptation of the present generation but can also be passed on to offspring through sexual reproduction. As a result, the unfavourable environmental conditions that parent plants face can create a type of inherited memory in future generations, which helps the offspring to quickly build resilience to adversity during their growth and development. This phenomenon, known as ‘stress-induced transgenerational memory’, demonstrates a close interplay with induced resistance, with the former laying the groundwork for the occurrence of the latter. This interrelationship holds significant implications for biological evolution and survival strategies. The stress-induced transgenerational memory in plants is closely associated with epigenetic factors such as DNA methylation, non-coding small RNA molecules, chromatin histone modifications, and chromatin accessibility [78].

In *Arabidopsis*, genome methylation is closely related to peach aphid feeding. Aphid feeding results in demethylation at hundreds of loci, resulting in the transcriptional activation of transposable elements. The deletion of methylation loci regulates the expression of genes, including those resulted to the plant immune response. By exploring the role of epigenetic factors in the aphid resistance of Arabidopsis mutants, it was found that the H3K9 metabolism mutant (*kyp*) showed increased resistance to peach aphid. The results of those studies indicate that DNA methylation plays an important role in the relaxation of epigenetic control and the associated regulatory mechanisms [79].

Studies on various organisms, such as *Arabidopsis* and rice, have detected transgenerational epimutations induced by multigeneration stress, underscoring the significance of DNA methylation as a molecular basis for transgenerational effects [80, 81]. The identification of the *Karma* gene locus in oil palm and its association with fruit cracking highlights the importance of DNA methylation in transposon activity and gene regulation [82]. To date, most research in this area has focused on elucidating the mechanisms and interactions between DNA methylation and gene regulation. Few studies have explored the regulatory mechanisms and genetic patterns underlying this phenomenon. This has limited the applications of DNA methylation to induce resistance to biotic stress in breeding programmes. Further research on the processes underlying transgenerational induction of resistance in the context of the plant–aphid interaction is warranted. If plants can pass on induced resistance to new generations through sexual reproduction, this is an exciting avenue for the production of more resilient crop varieties [83].

## Molecular breeding to generate aphid-resistant plants

Technological developments in molecular plant breeding have resulted in highly accessible breeding methods that are both rapid and precise [6]. Scientists and plant breeders can make rapid progress in the molecular design of aphid-resistant plant varieties by using a combination of population genetic analyses, multiomics techniques, and gene-editing tools such as CRISPR-Cas9.

### Plant breeding utilizing forward genetics approaches

Methodologies such as QTL mapping, GWAS (Genome-wide association study), and BSA (bulked segregant analysis) are indispensable tools for dissecting the intricate genetic architecture of complex traits. These tools make it possible to search for, and identify, candidate genes involved in plant resistance against aphids. Using various markers including AFLP (amplified fragment length polymorphism), SSR (simple sequence repeat), and SNP (single-nucleotide polymorphism) markers, genetic linkage maps have been created, and QTLs for aphid resistance have been identified. These types of analyses have been conducted for a wide range of plants, including important crop plants like wheat, maize, soybean, and cucumber, amongst others [9, 84].

QTL mapping and transcriptomic analyses of maize inbred lines revealed three candidate genes related to aphid resistance: Zm00001d035736, Zm00001d035751, and Zm00001d035767 [8]. In cotton, QTL mapping using the F_2_ generation population derived from hybridization between the aphid-resistant cultivar Xinluzao 61 and the aphid-sensitive cultivar Xinluzao 50 identified *GhLAC4-3,* encoding an enzyme involved in lignin synthesis, as a gene related to aphid resistance. Further virus-induced gene silencing (VIGS) analyses proved that this gene positively regulates aphid resistance in cotton [85]. Soybean aphid (*Aphis glycine* Matsumura) seriously threatens the yield of soybean. By crossing the aphid-resistant soybean strain E08934 with the aphid-sensitive soybean strain E00003, two aphid-resistant QTL_S_, Rag6 and Rag3c, were found in the constructed localized population. These two QTLs were proven to confer aphid resistance in no-choice tests [9]. Peach aphid is a severe pest in pepper production, and QTL mapping to identify genomic regions related to resistance to this aphid identified two QTLs: Rmpas-1, related to decreased aphid survival rate, and Rmprp-1, related to reduced aphid reproduction [86]. In cucumber, a major QTL related to resistance to cotton aphid was identified on chromosome 5, in a 0.31-Mb region containing 43 genes. Genes in this region with an LRR gene structure were identified as key candidate genes for aphid resistance [87, 88]. A QTL for aphid resistance was detected on chromosome 2 of pepper. This QTL significantly impacts aphid survival and reproduction, and has been finely mapped to a locus containing an LRR-RLK gene associated with plant resistance [86].

The combination of SSR and SNP marker genotyping has been used successfully for genetically mapping a novel aphid (*Aphis craccivora*) resistance locus in the cowpea breeding line SARC 1–57-2 [89]. This method has also been instrumental in introgressing resistance into elite cultivars through marker-assisted backcrossing.

With the development of high-throughput sequencing technology, GWAS and BSA methodologies have been used more widely for the exploration and mining of plant aphid resistance genes. GWAS identifies the presence of sequence variants known as SNPs throughout the genome of a plant, from which SNPs associated with aphid-resistant traits can be screened [90]. A GWAS for pea (*Pisum sativum*) resistance against a pea-adapted biotype and a non-adapted biotype of pea aphid (*A. pisum*) identified a genomic region on chromosome 7 conferring resistance to both biotypes. This region, the major-effect QTL *ApRVII*, included linkage disequilibrium blocks significantly associated with resistance to one or both of the aphid biotypes studied [91].

Compared with GWAS, BSA is a less costly technique for dissection of traits of interest. BSA can be used to target aphid resistance traits by selecting parents with extreme phenotypic differences to construct a family line. Whole-genome resequencing of the two parents and the two mixed pools of progeny with extreme aphid resistance trait phenotypes can then reveal sequence differences related to the trait [92]. In peach, BSA mapping of aphid-resistant and aphid-susceptible individuals in segregating progeny populations delimited Rm3 to a 160-kb interval containing 21 genes on chromosome 1. A chromosomal structural variation encompassing two novel TIR–NLR-class disease resistance (R) protein-coding genes was identified in that interval [93].

After QTL and candidate genes have been identified, multiomics analyses can provide details of the genetic control of aphid resistance in plants. For example, Wang et al. (2023) integrated QTL mapping with transcriptome and metabolome analyses, and detected three candidate genes associated with aphid resistance that function via the hypersensitive response [8].

### Plant breeding by genetic engineering

In the current landscape of molecular plant breeding for aphid resistance through genetic engineering, three principal strategies have emerged. The first involves introducing genes that allow plants to synthesize compounds detrimental to aphids, such as lectins or fusion proteins. The second strategy aims to decrease the attractiveness of plants to aphids. The third strategy is to enhance plants’ innate immunity against aphids [94].

Phytohemagglutinin is a carbohydrate-binding protein with a high binding affinity for specific glycans of glycoproteins and glycolipids on cell membranes, and it is harmful to aphids [95]. Transgenic cotton lines expressing an insect gut-binding lectin from *Sclerotium rolfsii* Sacc. showed resistance to sucking and chewing insects that was maintained in the T_1_ generation. In addition, populations of the aphid were reduced by ~69% and *Spodoptera litura* mortality reached 100% within 96 h [96].

Multiple sequences can also be introduced to improve aphid resistance. That is, sequences for several structural domains of different insecticidal proteins can be linked together to generate fusion proteins inside the transformed plants. These proteins are then transcribed and translated into individual polypeptide units in the host plant system. This fusion protein strategy has proven to be very effective and potent against phytophagous insects [97]. An example of this strategy is the fusion protein of lectins and protease inhibitors transferred into *B. juncea* by *Agrobacterium*-mediated transformation. Plants harbouring the transgene showed high expression levels of the fusion protein accompanied by resistance to phytophagous aphids, which had a survival rate of only 40% after feeding. This demonstrates the successful application of genetic engineering methods to improve resistance against aphids [98, 99].

In watermelon, knockdown of *VST1* by CRISPR-Cas9 technology and overexpression of *VST1* affected attractiveness to aphids and the degree of resistance to aphid attack. The results indicated that *VST1* is a negative regulator of aphid resistance in watermelon [100].

Molecular plant breeding has shown great potential for improving plant resistance to aphids. Ongoing progress in molecular plant breeding technologies is providing extensive opportunities for improving plant resistance to aphids. Population genetics and multiomics methodologies, together with advanced techniques such as CRISPR-Cas9, are allowing scientists and breeders to devise accurate and effective methods to generate new insect-resistant plant varieties.

Tools such as QTL mapping, GWAS, and BSA are useful for identifying key genes related to plant resistance to aphids. The development of genetic linkage maps using molecular markers makes it possible to precisely locate resistance loci. In addition, genetic engineering techniques can be used to introduce genes that allow plants to produce toxic substances that are detrimental to aphids, that decrease their appeal to aphids, and/or that strengthen their natural defence system, thereby generating more resilient plant varieties. These methods have been used to generate new lines with increased resistance to aphids in several crop species.

## Conclusions

In this review, we outline research progress on plant breeding for aphid resistance, focusing on the mechanisms and breeding tactics from five perspectives. First, we outline the three mechanisms of plant resistance to aphids: antixenosis, tolerance, and antibiosis. Then, each mechanism of aphid resistance is discussed in detail to explain the complexity of aphid resistance. The damage caused by aphids lies not only in their high reproductive rate, but also in their ability to transmit plant viruses. We discuss how aphids disrupt plant defensive responses by spreading viruses, as well as how various types of viruses affect plant development, aphid selection behaviour, and internal plant reactions. Many conventional crop varieties are not sufficiently aphid resistant to meet the demands for high-quality and high-yield agricultural outputs with minimal pesticide use. Therefore, there is a need to generate aphid-resistant cultivars. Wild resources can be a resource for the introduction of traits related to aphid resistance, e.g. genes encoding toxic compounds or those related to resistance responses. Although molecular breeding methods can be used to genetically modify original cultivated crop varieties, aphid-resistant wild species can also be *de novo* domesticated to meet the requirements of modern agricultural production. We discuss the applications of plant-induced resistance and transgenerational inheritance in biotic and abiotic stress, as well as their potential role in breeding for aphid resistance. Finally, we discuss the application of molecular breeding technology to produce aphid-resistant crop varieties for sustainable crop production. Over the past decade, significant progress has been made in crop domestication and breeding, including the construction of crop genome maps, functional gene studies, and the application of complex genetic information processing methods. The results of these analyses provide foundational information for efficient crop improvement whilst deepening our understanding of crop evolution mechanisms, offering solutions to future food security challenges.

## Data Availability

Not applicable.
